# Clinical utility of Phototest via teleneuropsychology in Chilean rural older adults

**DOI:** 10.1590/1980-5764-DN-2021-0082

**Published:** 2022-06-24

**Authors:** Nicole Caldichoury, Marcio Soto-Añari, Loida Camargo, María Fernanda Porto, Jorge Herrera-Pino, Salomón Shelach, Claudia Rivera-Fernández, Miguel Ramos-Henderson, Pascual Angel Gargiulo, Norman López

**Affiliations:** 1 Universidad de Los Lagos, Departamento de Ciencias Sociales, Osorno, Chile.; 2 Universidad Católica San Pablo, Laboratorio de Neurociencia, Arequipa, Perú.; 3 Universidad del Sinú, Facultad de Medicina, Cartagena de Indias, Colombia.; 4 Universidad de la Costa, Departamento de Ciencias Sociales, Barranquilla, Colombia.; 5 Florida International University, College of Medicine, Florida, USA.; 6 Universidad Nacional de San Agustín de Arequipa, Arequipa, Perú.; 7 Universidad Santo Tomás, Facultad De Salud, Centro de Investigación e Innovación en Gerontología Aplicada, Antofagasta, Chile.; 8 Universidad Nacional de Cuyo, Facultad de Ciencias Médicas, Departamento de Patología, Laboratorio de Neurociencias y Psicología Experimental, Mendoza, Argentina.

**Keywords:** Neuropsychological Tests, Telemedicine, Cognitive Dysfunction, Rural Population, COVID-19, Testes Neuropsicológicos, Telemedicina, Disfunção Cognitiva, População Rural, COVID-19

## Abstract

The COVID-19 pandemic has shown the need for neuropsychological care for older adults with memory complaints in different contexts, including rural areas or areas with difficult access.

**Objective:**

This study aimed to analyze the clinical utility of the Phototest, through telemedicine, to identify mild cognitive impairment in rural older adults with memory complaints, during the COVID-19 pandemic.

**Methods:**

We performed a cross-sectional, case-control, and clinical utility comparison of brief cognitive tests (BCTs). The sample included 111 rural elderly people with mild cognitive impairment (MCI) and 130 healthy controls from the Los Lagos region, Chile. The instruments adopted were modified Mini-Mental State Examination (MMSEm) and adapted version of the Phototest (PT) for Chile.

**Results:**

To identify mild cognitive impairment, using a cutoff score of 27-28 points, the Phototest showed a sensitivity of 96.6% and a specificity of 81.8%; indicators superior to those of the MMSEm.

**Conclusions:**

The Phototest is more accurate than the MMSEm in identifying cognitive alterations in rural older adults with cognitive memory complaints through telemedicine. Therefore, its use in primary care is recommended in order to perform early detection of preclinical cognitive alterations in mild cognitive impairment or neurodegenerative diseases.

## RESUMO.

A pandemia de COVID-19 mostrou a necessidade de cuidados neuropsicológicos para adultos idosos com queixas de memória em diferentes contextos, incluindo áreas rurais ou áreas de difícil acesso.

**Objetivo:**

Analisar a utilidade clínica do Phototest, por meio da telemedicina, para identificar uma leve deficiência cognitiva em adultos idosos rurais com queixas de memória, durante a pandemia de COVID-19.

**Métodos:**

Realizamos uma comparação transversal, caso-controle e utilidade clínica dos testes cognitivos breves. Amostra: Cento e onze idosos rurais com deficiência cognitiva leve (DCL) e 130 controles saudáveis da região de Los Lagos, Chile. Instrumentos: Minimental modificado (MMSEm) e versão do teste fotográfico (PT) adaptada para o Chile.

**Resultados:**

Para identificar a DCL, usando pontuação de corte de 27-28 pontos, o Phototest mostrou sensibilidade de 96,6% e especificidade de 81,8%; indicadores superiores aos do MMSEm.

**Conclusões:**

O Phototest é mais preciso que o MMSEm para identificar, por meio da telemedicina, alterações cognitivas em adultos idosos rurais com queixas de memória cognitiva. Sendo assim, seu uso na atenção primária é recomendado para realizar a detecção precoce de alterações cognitivas pré-clínicas em DCL ou doenças neurodegenerativas.

## INTRODUCTION

Due to the Coronavirus pandemic, several medical and hospital care services focused on the elderly were suspended to control the viral spread and reduce mortality^1^. One of these services was routine clinical and neuropsychological assessments related to cognitive impairment (CD), given their nature in terms of the interpersonal contact involved^2^. Unfortunately, during quarantine, there was evidence that medical conditions such as diabetes and hypertension^3^, considered risk factors for CD^4^, worsened and that neuropsychiatric symptoms and the risk of CD increased among the elderly^5^. Therefore, the field of neuropsychology had to quickly evolve and adapt, by incorporating telehealth or teleneuropsychology (TNP) assessments to continue providing cognitive assessment and monitoring services to the elderly^6^ ^,^ ^7^.

There is a growing literature that supports TNP as a feasible and reliable way to conduct neuropsychological tests and assess the cognitive status of elderly, given the barriers that this technology allows to overcome^8^. During the global COVID-19 pandemic, TNP has proven to be useful in detecting and monitoring CD in the elderly^9^ ^,^ ^10^. The available literature shows that TNP can provide reliable and valid assessments^11^ ^,^ ^12^, reaching geographically distant populations to identify CD^13^ ^,^ ^14^, thus becoming a tool of major clinical value to address the current situation of confinement and social distancing, especially in primary health care^15^ ^,^ ^16^.

However, the development of TNP in Latin America (LA) is still in its incipient, and the assessment of CD with the brief instruments through telehealth formats in rural seniors has not been conducted in the region^17^. Few studies have analyzed the reliability and validity of neuropsychological tests in the telehealth context among Latin or Hispanic people^18^; mainly in preclinical phases of dementia, such as mild cognitive impairment (MCI).

Likewise, several of the brief cognitive tests (BCTs) available in the region have experienced problems of general clinical utility, low sensitivity, and specificity, especially in early stages of CD such as in MCI^19^ ^,^ ^20^. The difficulties of diagnostic utility have been described in a population with low schooling^21^ ^,^ ^22^; in addition, most of the research that has validated BCTs has been carried out in an urban population^19^.

In contrast, the Phototest (PT)^23^ has demonstrated its usefulness in primary health care centers, since it does not use pencil or paper, which facilitates its performance and evaluation, especially in people with lower educational levels or in those who are illiterate^24^ ^,^ ^25^. It has a higher sensitivity and specificity than traditional tests with respect to dementia and MCI^26^ ^,^ ^27^ and has shown to be more effective and cheaper than the Mini-Mental State Examination (MMSE) in multiple studies^27^ ^,^ ^28^ ^,^ ^29^. Finally, given its structural and managing characteristics, it is believed to be easily adapted to virtual assessment formats.

Therefore, considering that the percentage of rural seniors in LA is higher than their urban counterparty^29^, they have fewer years of schooling, higher disease burden, restricted access to specialized medical controls, and a lower probability of having contracted health insurance, it is necessary to have MCI-sensitive instruments that can be applied in rural settings through TNP. Therefore, our objective was to analyze the clinical utility of the PT to detect MCI in older adults in rural areas of Chile during the SARS-CoV-19 pandemic, using TNP.

## METHODS

### Design

This is a cross-sectional, case-control study of clinical utility analysis of BCTs, using TNP, in a non-probability sample of 111 rural elderly with MCI and 130 controls. It was carried out during the second half of 2020, i.e., during national quarantine, due to the global pandemic of the COVID-19.

### Participants and procedure

The initial sample was composed of 354 rural seniors, aged 65 years or older, who attended the annual checkup part of the Preventive Medicine Program for the Older Adult (EMPAM for its acronym in Spanish). This is a national program of the Chilean Ministry of Health that is carried out in all primary care centers in the country^30^; it is implemented by an interdisciplinary team (e.g., medicine, nursing, kinesiology, and psychology). It consists of a comprehensive, periodic, and follow-up clinical evaluation to detect factors that may affect the health, autonomy, and independence of this population. In this annual medical control, the Functional Examination of the Elderly (EFAM) is applied, which is used to predict the loss of functionality of the elderly. This instrument allows classifying the subjects according to the degree of functionality: self-valent or autonomous, self-valent with risk, and those at risk of dependence. The EFAM includes a clinical and sociodemographic record, anthropometric measurements (e.g., blood pressure, pulse, weight, height, and body mass index, waist circumference, and physical activity), together with a functional assessment [Barthel test^31^, Pfeffer and risk of falls using the unipodal station test^32^ ^,^ ^33^, and the Timed Up and Go (TUG)^34^], cognitive assessment [abbreviated Mini-Mental or MMSE-EFAM (MMSE-EFAM)^35^ ^,^ ^36^], and mood evaluation [Yesavage depression scale^37^]. The EFAM^30^ consists of two parts. In part A, the functional aspects are evaluated, and in part B, the cognitive and emotional dimensions are examined; in addition, the aspects that affect the mental health and functionality of the elderly person are also examined. The results allow the identification of loss of functionality, health problems, suspicion of depression, and CD. This information guides the professional in assessing the cognitive and functional status of the elderly person.

In the context of this annual evaluation, 354 older adults were assessed at a family health center (CESFAM) in a rural sector of the Los Lagos region (southern Chile). Of these, 181 reported having memory problems and 173 did not report such problems. All patients were evaluated with the EFAM, together with a CDR clinical interview and medical assessment. The assessment was performed through the Zoom platform, except for the anthropometric measurements, the TUG, and the unipodal station test, which was performed in person at the rural doctor’s office, during the patient’s first visit.

In the first group, with memory complaints, 111 participants were identified with MCI, but autonomous or functional, whereas, in the second group, without memory complaints, 130 participants were confirmed as autonomous or functional elderly, without cognitive problems. In both the groups, clinical and psychometric criteria were applied to confirm the MCI group and the healthy control (HC) group. To identify the subjects as MCI and HC, first, the results of the EFAM functional tests (i.e., Barthel Test, Pfeffer, TUG, and unipodal station test) were considered. In the case of the MCI group, the result of the functional assessment should classify the patient as a self-sufficient or autonomous older adult, according to the first part of the EFAM. Although individuals with very mild functional failure in instrumental activities of daily living were included. Then, in the second part of the EFAM, the patient had to obtain CD scores on the abbreviated MMSE or MMSE-EFAM (≤13 points). In addition, the score on the CDR had to be 0.5. In contrast, HC had to be classified as self-valent or autonomous on the EFAM, obtaining high scores on the abbreviated MMSE (≥14 points) and classified by the CDR as 0.0.

After being evaluated with the EFAM, 113 subjects were excluded from the study for presenting functional problems, suspected dementia, depression, or voluntarily withdrew from the study. But they were redirected to the psychosocial care program. Finally, the presumptive diagnosis made by primary care professionals was reviewed and validated by a neuropsychologist and an expert neurologist. ­Subsequently, two groups were established, one consisting of older adults with MCI (MCI=111) and healthy controls (HC=130). Then, all patients underwent a TNP assessment, where the modified MMSE (MMSEm)^38^ and the PT^39^ were administered.

### Instruments and digital platform

The PT ^23^ is a short cognitive test that can be used for free under a Creative Commons license and is very suitable for primary care centers. This test does not use pencil and paper and is easy to administer and score, especially for people with low educational levels. The PT comprises three parts (Annex 1 Supplementary Material): (1) a naming task (30-60 seconds), with six color photographs of common objects in prototypical position (i.e., card, car, pear, trumpet, shoes, and spoons); (2) a verbal fluency test (names of people: men and women separately, 30 s each); and (3) a free recall task and recall facilitated by the cues, using the six objects of the naming test (30-60 s). The administration of the test lasts for approximately 3 min.

The team of health center professionals was trained in virtual telehealth programs and TNP procedures; in addition, they were trained by neurologists and neuropsychologists with expertise in cognitive pathology and detection of CD and dementia. They were trained in the use of clinical criteria, clinical interview (CDR), and cognitive instruments, including the MMSEm and PT. For the TNP assessment of subjects with MCI and HC, the modified Mini-Mental^38^, the adapted version of the PT for Chile^39^, and a demographic card was included. The Zoom platform was used for real-time neuropsychological assessment. The MMSE tasks that required writing and drawing were assessed remotely using pencil and paper. Patients were asked to show their drawings to the camera for the practitioner to examine. The evaluator graded, allowing the family member, companion, or caregiver to assist them with the use of the computer and webcam. The PT was projected on the virtual platform and did not require patients or their companions to manipulate the test. International clinical guidelines and recommendations for TNP assessments in times of pandemic were followed^2^ ^,^ ^12^.

### Statistical analysis

A descriptive analysis of the demographic characteristics and the results of cognitive tests was performed on the groups analyzed. A comparison between groups was performed using Student’s t-test, and an analysis of the clinical utility of the instruments was performed using ROC curves. Sensitivity (Sn) and specificity (Sp) values were calculated along with the cutoff scores suggested in the Chilean literature for the MMSEm (≤21=CD; ≥22=HC)^38^ and the PT (28-29=MCI; ≥30=HC)^23^. SPSS version 25 was used.

### Formal aspects

The procedures performed in the present study complied with the ethical standards of the pertinent national committees and institutions on human experimentation and the Declaration of Helsinki 1975, which was revised in 2008. All participants were informed about the nature of the study and signed consent. This study was approved by the Institutional Ethics Committee of Universidad Santo Tomás, in Chile (CEC UST Nº 15), and of Universidad de La Costa, in Colombia (Act No. 092). The research is part of an international and multicenter study.

## RESULTS

**Figure 1.**
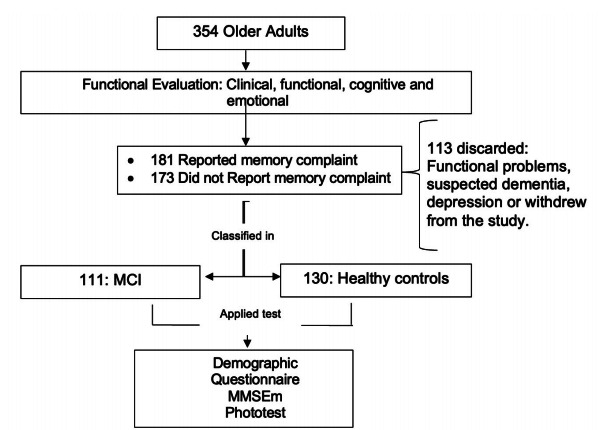
Study flow diagram.

The flow diagram of the participants is shown in ­Figure 1. As can be seen in Table 1, no significant differences were found in terms of age and schooling among participants. In contrast, gender and average cognitive test performances between the study groups did show significant differences. Subjects without cognitive complaints showed better cognitive performance.

**Table 1.** Demographic characteristics and results of the cognitive tests of the participants.

Number		MCI		HC		Student’s t /χ^2^	
		M	SD	M	SD	F	p
		111		130			
Age		70.342±9.51		71.12±7.85		25.40	0.239
Gender	Women	71		81		2.17	0
	Men	40		49			
Years of schooling		6.72±2.45		6.91±3.00		6.18	0.644
MMSE		23.82±2.96		25.83±3.77		147.80	0.00
Phototest		26.82±3.82		36.16±6.28		100.34	0.00

MCI: mild cognitive impairment; HC: healthy control; M: mean; SD: standard deviation; MMSE: Mini-Mental State Examination.

A comparison between the PT and the MMSEm was performed among participants. Table 2 shows the aROC along with the cutoff score, in which Sn and Sp are best balanced for CD. The PT used a cutoff score of 26/27 points and exhibited a higher Se=96.6 and Sp=81.8 than the MMSE (Se=56.9; Sp=72.7). Similarly, ROC curve analysis showed that the PT has a higher area under the curve (AUC=90%) than the MMSEm (AUC=69%) in the MCI group.

**Table 2.** Sensitivity and specificity of the Mini-Mental State Examination and the Phototest.

Participants	Test	Cutoff	aROC	Se	Sp
MCI vs. HC	Phototest	27-28	0.90 (0.80-0.99)	96.6 (0.85-0.99)	81.8 (0.71-0.91)
	MMSE	≤ 21	0.69 (0.54-0.83)	56.9 (0.27-0.86)	72.7 (0.61-0.84)

aROC: curve area; Se: sensitivity; Sp: specificity; MCI: mild cognitive impairment; HC: healthy control.

## DISCUSSION

The MMSE is the gold-standard test for the detection of CD^40^ ^,^ ^42^. Despite this, difficulties have been reported with this instrument for several years. Test administration is not standardized for TNP formats in all LA countries, where the cultural, educational, and socioeconomic characteristics of the patient may bias scores^42^. This test does not measure executive function as it can detect only moderate or advance dementia^43^; and it is also not sensitive to MCI^44^ ^,^ ^45^, early stages of Alzheimer’s-type dementia, and non-Alzheimer’s-type dementias^46^. As a pencil and paper task, the MMSE conducted through telehealth means requires the use of complex digital platforms or the user to interact with other systems to complete the assessment^47^. Additionally, the use of the MMSE in primary health care is limited due to its long completion time and educational bias; consequently, it cannot be applied to illiterate people since several of its items require verbal and writing skills.

Moreover, the PT is a very brief test (<3 min) that can be used in primary health care and clinical contexts that have very limited time and large volumes of patients seeking treatment^48^. It can be applied to illiterates and is not affected by the level of education^47^. In addition to memory, the PT assesses executive function, and it is a test that has no ceiling effect^20^. It has proven to be more effective, economical, and efficient than the MMSE in identifying dementia in primary health care and can differentiate MCI from dementia^25^ ^,^ ^26^ ^,^ ^49^. In this study, the PT with a cutoff score of 26/27 points exhibited excellent psychometric indicators (Sn=96.6 and Sp=81.8), for identifying MCI; far superior to those of the MMSE (Se=56.9; Sp=72.7) revealing greater clinical utility (PT: AUC=90% vs. MMSE: AUC=69%).

Recent studies have reported the superiority of the PT in identifying CD in general and MCI in various health contexts^29^ ^,^ ^50^, including their identification in illiterate or low-schooling subjects^51^ ^,^ ^52^, showing that PT was one of the most accurate tests to detect suspected CD, especially in patients with lower levels of education or in those coming from different cultural backgrounds. Nevertheless, in a systematic review of BCTs for early detection of dementia in LA elders, Custodio et al.^53^ reported difficulties in a broad spectrum of BCTs, including MoCA, ACE-R, and the Ineco Frontal screening. The authors of this study argued that most of the tests required cultural adjustment and different cutoff scores depending on educational level, while others were to be analyzed in populations with low levels of education. This review did not take into account the analysis of the PT. But in the same year, Burke et al.^54^ analyzed 10 cognitive tests for dementia in the Spanish-speaking population, using the PT and concluded that this instrument presented the highest statistical indicators to detect dementia and MCI and was the most appropriate to be applied in contexts of low levels of education and literacy. We believe that the origin of these discrepancies may be the lack of studies analyzing the properties of the PT in LA. The fact is that the PT is receiving more and more support, given its diagnostic accuracy and its usefulness in detecting CD in people with a low level of education or illiteracy^25^ ^,^ ^52^.

Another interesting result was related to the characteristics of the digital platform used and the effectiveness of remote neurocognitive assessment, to identify CD in the participants of this study. Since the PT is not a pencil and paper task and does not require the subject to manipulate the test, its inclusion in the digital platform was easy. Also, the PT has several advantages over other available BCTs. The test (face-to-face or remote) starts with a naming task that includes a slide with six images, which is shown to the patient. The developers of this instrument have created several slides to perform the assessment, thus reducing the learning effect and diagnostic errors of the instrument (http://www.fototest.es/). Additionally, the application of the test is notably fast^55^, easy to score and interpret, and did not generate rejection among the participants of this study. On the contrary, MMSEm usage experience through virtual assessment resulted in several participants rejecting the orientation items, arguing they had been disrespected. In other cases, family or technological support was needed to complete the writing and drawing tasks. Therefore, some cases required the training of patients or companions on the answering of the test. In other cases, the application of the MMSEm could not be completed by low-schooling or illiterate subjects, thus producing rejection and several people desist from participating in the study.

Therefore, despite the evidence supporting the assessment of dementia in vulnerable populations through telemedicine^15^ ^,^ ^56^ ^,^ ^57^ ^,^ ^58^ ^,^ ^59^, some conditions must be met. The main recommendations considered in our study was training in TNP; for professionals to develop competencies to manipulate telehealth platforms, ethical issues such as informed consent, and biosafety procedures; and ability to address technical problems, connectivity, and social communication strategies and empathy through a screen^2^. In addition, the recommendations of the working group for the practice of TNP in LA^60^ were considered. These focused on the actions during the PT and MMSE administration procedure. It was verified that the patients had the necessary materials for the assessment (e.g., pen, pencil, and paper), the presence of a companion or facilitator, when appropriate, and the technological requirements^60^.

Therefore, since care should be provided to confined patients in rural areas of Chile, where access to medical care is limited^60^, our working group designed a strategy for TNP assessment. First, to use cognitive tests that could be used through virtual platforms and that the cognitive tests available for use using video technologies were fast, efficient, and reliable^12^, they should be adapted and implemented in culturally diverse populations, with low educational levels or illiterate^61^. In this order of ideas, the PT would meet the conditions to be administered to detect CD and dementia in elderly people with a low educational level or illiterate through TNP. This would be the first study that supports the use of PT through the TNP in LA, although the computerized version of the RUDAS 65 was recently analyzed in Peru and reported adequate psychometric indicators to discriminate CD and dementia in populations with heterogeneous educational levels, illiterate, and rural^19^ ^,^ ^62^ ^,^ ^63^.

This is pioneering research in LA, although it has some limitations. The authors of this study believe that follow-up studies are needed to gather evidence in favor of the PT using videoconferencing technologies for remote neuropsychological assessments. However, geographical and confinement limitations, in addition to limited access to professionals with expertise in cognitive pathologies, highlight the relevance of rapid TNP assessments. On the other hand, it would have been interesting to have compared the performance of this sample in urban populations. Unfortunately, this was not possible due to the sanitary measures of confinement. It was also not possible to analyze in depth the impact of quarantine or social isolation on the mental health and cognition of the participants. During the functional assessment (EFAM), subjects who exhibited significant symptoms of mood alterations, or those whose symptoms were reported by their caregivers or relatives, were excluded from this study and referred to the psychosocial care program. Questions about the emotional situation of the elderly during confinement were not included in the TNP assessment.

In addition, it was not possible to incorporate extensive neuropsychological assessments or to have complete cognitive profiles. Similarly, it was not possible to specify the type of MCI, due to logistical problems, bandwidth, and the complexity of applying extensive neuropsychological assessments remotely through these virtual environments. Recent reports stress that such connection-related difficulties can affect processing speed tasks. The low resolution of the screens can affect the discrimination of colors present in the tests. Finally, the quality of the audio can lead to loss of information or interruption^18^. These situations were addressed through the recording of the assessments and subsequent reviews.

Thus, PT proved to be more accurate in identifying MCI in elderly people in rural Chile using TNP compared with the gold-standard measure. Its use is recommended in primary health care contexts to detect preclinical cognitive alterations, such as MCI.
